# An apolipoprotein B100 mimotope prevents obesity in mice

**DOI:** 10.1042/CS20150423

**Published:** 2015-12-09

**Authors:** Hyo Joon Kim, Hee Jong Lee, Jung Soon Choi, Jemin Han, Ji Young Kim, Hyun Kyun Na, Hae-Jung Joung, Young Sik Kim, Bert Binas

**Affiliations:** * Department of Molecular & Life Science, College of Science & Technology, Hanyang University (ERICA), Ansan, 426-791, Republic of Korea; † SJ Biomed Inc., HBI 604, 55 Hanyangdaehak-ro, Ansan, 426-791, Republic of Korea; ‡ Pathology, Korea University Medical School, Ansan Hospital, 123 Jeokgeum-ro, Danwon-gu, Ansan, 425-707, Republic of Korea

**Keywords:** ApoB100, high-fat-diet-induced obesity, humoral immunity, mimotope

## Abstract

Although apolipoprotein B100 (ApoB100) plays a key role in peripheral fat deposition, it is not considered a suitable therapeutic target in obesity. In the present study we describe a novel ApoB100 mimotope, peptide pB1, and the use of pB1-based vaccine-like formulations (BVFs) against high-fat diet (HFD)-induced obesity. In HFD- compared with chow-fed adolescent mice, BVFs reduced the 3-month body-weight gains attributable to increased dietary fat by 44–65%, and prevented mesenteric fat accumulation and liver steatosis. The body-weight reductions paralleled the titres of pB1-reactive immunoglobulin G (IgG) antibodies, and pB1-reactive antibodies specifically recognized native ApoB100 and a synthetic peptide from the C-terminal half of ApoB100. In cultured 3T3L1 adipocytes, anti-pB1 antibodies increased lipolysis and inhibited low-density lipoprotein (LDL) uptake. In cultured RAW 264.7 macrophages, the same antibodies enhanced LDL uptake (without causing foam cell formation). These findings make ApoB100 a promising target for an immunization strategy against HFD-induced obesity.

## Abstract (precis)

Immunization with an ApoB100 mimotope prevents high-fat-diet-induced obesity in mice. Antibody titres parallel the weight decrements. *In vitro* data implicate increased lipolysis and reduced lipoprotein uptake by adipocytes, as well as increased uptake and metabolism of native lipoprotein by macrophages.

## CLINICAL PERSPECTIVES

• Although ApoB100 is an important player in peripheral lipid deposition, it has not yet been considered as a target for preventing HFD-induced obesity.
• We show that, in a vaccine-like formulation, an ApoB100 peptide mimotope prevents HFD-induced obesity in mice. Mechanisms may involve antibody-mediated inhibition of lipoprotein uptake and LDL-dependent inhibition of lipolysis, as well as opsonization-stimulated lipoprotein uptake by macrophages (no foam cell formation).
• As the human context appears even more favourable, the approach looks promising for the treatment of human obesity.

## INTRODUCTION

Apolipoprotein B100 (ApoB100) and ApoB48 are the full-length and C-terminally truncated versions of ApoB, an essential constituent of several classes of lipoproteins that are secreted by the liver and intestine [1]. In each of these lipoprotein particles, a single ApoB molecule provides a structural frame and, depending on the ApoB version and particle context, may interact with cell-surface receptors or lipases. In rodents and humans, ApoB48 vastly dominates ApoB100 in the intestinally derived lipoproteins (chylomicrons and their remnants). By contrast, human liver produces only ApoB100, whereas rodent liver produces a mixture of ApoB100 (slightly dominating) and ApoB48 [2]. The primary ApoB-containing lipoprotein secreted by liver is the very-low-density lipoprotein (VLDL), which in the circulation is converted into smaller intermediate-density lipoprotein (IDL) and low-density lipoprotein (LDL) particles while releasing fatty acids.

Given that VLDL and chylomicrons play central roles in the peripheral deposition of liver- or gut-derived fatty acids [3], the ApoBs could be considered as potential therapeutic targets in obesity. Indeed, it was recently shown that a partial reduction in ApoB48 levels in chow-fed rabbits (by RNA interference directed against Apobec1, ApoB mRNA-editing enzyme, catalytic polypeptide 1) led to a leaner and apparently healthier phenotype [4]. It remains to be seen whether such an approach can truly avoid the adverse effects associated with inborn deficiency of intestinal ApoB [5].

However, there is little, if any, support for the idea of using ApoB100 as a target for an anti-obesity treatment. Although VLDL overproduction is common in obesity, it is usually discussed as a consequence rather than a cause [6]. We are not aware of any hard evidence showing that the altered production of ApoB100/VLDL affects body weight. It was observed that the transgenic overexpression of human ApoB100 leads to production of triacylglycerol-rich ‘VLDL-like’ LDL, but altered body weights were not reported [7,8]. Conversely, human mutations that reduce or truncate ApoB100 in the C-terminal half (leaving ApoB48 intact) generally do not reduce body weight; in fact such mutations are more often associated with being overweight [9]. Perhaps the strongest argument against the idea of considering ApoB100 as an anti-obesity target is the observation that mice that do not produce hepatic ApoB and VLDL (due to liver-specific deletion of microsomal triacylglycerol transfer protein or MTTP) did not show an altered fat tissue mass even under a high-fat diet (HFD) [10].

Thus, the genetic studies have not encouraged the idea of ApoB100 as a primary target in obesity therapy. Moreover, as large multidomain molecules, the ApoBs (especially ApoB100) are involved in many interactions that are not necessarily related to triacylglycerol deposition [11,12]. In fact, most work on ApoBs is instead focused on their important and well-established roles in atherosclerosis. In this latter context, some laboratories have developed vaccines based on ApoB-derived peptides [13].

In the present study, we postulated that a similar immunization approach may reveal or unleash obesity-related roles of ApoB(s) that were not, or cannot be, revealed or unleashed by the genetic approach. Rather than directly using the ApoB amino acid sequence, however, we opted for a mimotope approach, which we expect to cause fewer safety concerns. We screened a phage peptide display library with monoclonal anti-ApoB100 antibodies and thereby discovered an ApoB100 mimotope peptide that elicits a specific antibody response against ApoB100 (but not ApoB48) and a potent protection against diet-induced obesity.

## MATERIALS AND METHODS

### Antigenic peptides

The coding sequence of peptide pB1 (RNVPPIFNDVYWIAF) was cloned in tandem into the expression vector pQE30 (Qiagen). We produced three configurations. The first, called B4, was a tandem of four pB1 units ([His tag]-[B1-linker]_4_, 86 amino acids). In the second configuration, hepatitis B viral peptide pre-S2, which contains a helper T-cell epitope [14], was added, resulting in peptide B4T ([His tag]-[B1-linker]_4_-T, 147 amino acids). The third configuration, called B4TB2 (181 amino acids), was identical to B4T except for two more [B1- linker] units. All peptides were produced in *Escherichia coli* by induction (4 h) with 1 mM IPTG, followed by His-tag affinity purification using open column chromatography.

### BVFs

The pB1-based vaccine-like formulations (BVFs) were prepared by mixing the antigenic peptides with sterile PBS and adjuvant, shaking at 4°C overnight and storing at 4–8°C. B4 was conjugated to ovalbumin (OVA) and mixed with Freund's adjuvant, resulting in ‘B4-OVA’. For B4T and B4TB2, we used 4% (v/v) Rehydragel HPA (Rehis Inc.; catalogue number 21645-51-2) as adjuvant. Vaccine, 0.1 ml, containing 0.05 mg of peptide (mice), 0.1 mg of peptide (rats) or no peptide (placebos) was injected intraperitoneally at the time points detailed in Table 1 (usually three injections at 2-week intervals).

**Table 1:** Peptide effects on rodent body weights over 12 weeks (week of first peptide injection till 12th week thereafter). The injection protocols indicate the weeks at which peptide injections were made (starting from the 8th week of age). Dietary fat contents (caloric contributions) were 10% for Chow and 60% for HFD.

Experiment	Animals	Diet/treatment	Injection protocol	Body weight (g) change over 12 weeks mean±S.E.M.	Peptide effect: Inhibition of HFD-caused weight increment (%)
		Chow		16.3±1.8	
1	ICR (♂)	HFD/GTG/Vehicle	0-2-4	40.6±2.8	
	n=6	HFD/GTG/B4-OVA		24.8±1.7**	65.0
		Chow		7.4±0.7	
2	C57/BL6 (♂)	HFD/Vehicle	0-2-4-6	20.7±0.7	
	n=6	HFD/B4T		13.5±0.4†*	54.1
		HFD/B4TB2		14.9±0.7**	43.6

*p<0.001 (HFD/Vehicle compared with HFD/B4T); **p<0.005 (HFD/Vehicle compared with HFD/GTG/B4-OVA or HFD/B4TB2); †three independent experiments.

### Animals and diets

Mice (strains C57BL/6, Balb/c and ICR) and rats (Sprague–Daley strain) were from SLC, Inc. The animals were kept in a temperature- and light-controlled room (25°C; 12 h light/12 h dark) and allowed free access to water and food. Food was purchased from Research Diets, Inc. and was either low (10 kcal% fat by calories, # D1250B; ‘chow’) or high (60 kcal% fat by calories, # D12492; HFD) in fat. Food intakes were determined twice a week in individual cages by subtracting the residual weight from the initial amount after a 24-h interval at 4:00 p.m. All animal experiments were approved by the institutional review board.

### Antibodies

Antibodies used study are summarized in Supplementary Table S1.

### Protein concentrations

Protein concentrations were determined using the BCA assay from Pierce.

### Measurements of mRNA

RNA was prepared from the spleens of mice killed at day 2 after the third boosting injection, and interleukin-4 and interferon-γ mRNA levels were quantified using real-time PCR and primer pairs: 5′-CCTGCTCTTCTTTCTCGAATGT-3′/5′-CACATCCATCTCCGTGCAT-3′ and 5′-TCAAGTGGCATAG-ATGTGGAAGAA-3′/5′-TGGCTCTGCAGGATTTTCATG-3′.

### Dot-blot experiments

Synthetic peptides (Peptron) and proteins were dissolved in DMSO at 1 mg/ml and diluted in PBS to the amounts given in the figures (per 50 μl). A 0.2-μm PVDF membrane (Millipore) was mounted into a 96-well Bio-Dot Microfiltration Apparatus from Bio-Rad Laboratories and pre-wetted with TBS, pH 7.5. Peptide or protein solutions (50 μl) were spotted on to the membrane and allowed to slowly (30–40 min) filter through on to an absorption pad. The membrane was then stained with Ponceau S solution to verify completeness of the transfer. For immunostaining, the membrane was blocked for 1 h at 37°C in 5% (w/v) non-fat dried skimmed milk powder in T/TBS (0.1% Tween in TBS), incubated overnight at 4°C with the primary antibody (1:500 dilution in T/TBS) or serum (1:200 dilution in T/TBS) indicated in the figure legend, washed with T/TBS three times, incubated with the secondary antibody [goat anti-mouse immunoglobulin G (IgG) horseradish peroxidase (HRP, Sigma, A0168) diluted 1:10000, in T/TBS] for 1 h at 37°C, washed again three times with T/TBS, and finally developed using the ECL Prime Western Blotting Detection Reagent (GE Healthcare).

### Western blot and ELISA

SDS/gel electrophoresis and Western blotting were performed according to standard procedures with the protein amounts indicated. Stated lipoprotein quantities refer to protein contents in both Western blotting and ELISA experiments. Indirect ELISA was performed in 96-well plates with a standard protocol using 500 ng of lipoprotein per well, 0.05% casein for blocking, and a sequential incubation with the given dilution of anti-B4T antibody (1 h at 37°C) and HRP-conjugated anti-mouse IgG (0.1 μg/ml; Sigma, A0168). ApoB100 was purchased from Calbiochem (catalogue number 178456). Human LDL and VLDL were prepared from human sera (Sigma, H1388) by density gradient ultracentrifugation [15].

### Uptake of radioactive dietary fat

Sprague–Daley rats were maintained with the regular chow, and immunizations were performed at 8, 10, 12 and 35 weeks of age. Some 4 days after the final injection, the rats were starved for 24 h, re-fed with chow for 12 h, and 5 μCi/head of glycerol tri[1-^14^C]oleate (GE Healthcare, CFA258) was administered with an intragastral catheter. After 3, 6, 10, 15 and 24 h, homogenized tissue samples were obtained for radioactivity measurements with a Liquid Scintillation Analyzer (Tri-Carb 2910TR, PerkinElmer).

### *In vitro* lipolysis experiments

*In vitro* lipolysis was measured in 3T3L1-derived adipocytes and primary adipocytes. The 3T3L1 preadipocytes were plated into 96-well plates, grown to confluence and differentiated [16]. They were then pre-incubated for 24 h with or without LDL or antibodies at 160 μg/ml each, followed by 2 h in the absence or presence of 0.1 mM noradrenaline (norepinephrine), and lipolysis was assessed by the measurement of glycerol release using a kit (Zen-Bio, Inc., LIP-1-L1). The primary adipocytes were isolated by collagenase digestion [17], seeded at 1×10^6^ cells/well into six-well plates, and incubated for 2 h in the absence of presence of 0.1 mM noradrenaline. Lipolysis was assessed by measuring glycerol release, using a kit from BioAssay Systems (EnzyChrom Adipolysis Assay Kit, EAPL-200). Generally, one assay was done with cells from one fat pad, but, after chow feeding, two to three epididymal fat pads had to be pooled.

### *In vitro* LDL uptake experiments

The 3T3L1 mouse preadipocyte and RAW 264.7 mouse macrophage cell lines were obtained from the American Type Culture Collection and maintained in Dulbecco's modified Eagle's medium (DMEM)/10% FBS. Before the experiments, 3T3L1 cells were transferred into six-well plates, grown to confluence and induced to differentiate into adipocytes [16], whereas the macrophages were seeded into six-well plates (1×10^5^ cells/ml) and used 12 h later. To assess LDL uptake, the cells were incubated for 24 h in DMEM/10% FBS supplemented with 50 μg/ml of human LDL, oxidized human LDL (oxLDL) or human LDL pre-incubated for 24 h at 4°C with the indicated antibodies (80 μg of antibody+40 μg of LDL in a total volume of 3 ml). Both unlabelled and 1,1′-dioctadecyl-3,3,3′,3′-tetramethyl-indocarbocyanine percholate (DiI)-labelled LDL and oxLDL (Biomedical Technologies, Inc.) were used. After incubation, the cells were photographed (with and without fluorescence), subjected to fluorimetry using a Synergy HT instrument (BioTek), stained with Oil Red O, or suspended in ice-cold PBS for flow cytometry. Samples of 1×10^4^ cells were analysed using FACSCalibur (BD Bioscience) [18]. In some experiments, analysis was preceded by an additional 24-h incubation step which, unlike the first incubation, was performed in the absence of LDL, oxLDL and antibody.

### Histology and evaluation

Haematoxylin and eosin (H&E)-stained tissue sections were prepared by a contract research organization and then assessed by a professional veterinary pathologist from the same company (Chemon, Inc.). The pathologist was not informed about the experimental treatment of the mice.

### Statistics

*P* values were determined via ANOVA and Bonferroni's multiple comparison test. Differences were considered significant for *P*<0.05. Error bars in the figures show S.E.M.

## RESULTS

### Discovery of an ApoB100 mimotope that prevents obesity

Using two monoclonal antibodies raised against human ApoB100 [19], we screened a phage display library [20,21]. One antibody retrieved eight peptides at different frequencies, of which two hits, including the most frequent one, were identical with the two peptides retrieved with the other antibody (see Supplementary Table S2). The most frequent hit, peptide RNVPPIFNDVYWIAF, was named pB1. This sequence does not show any similarity to human or mouse ApoB100 (using the National Center for Biotechnology Information's protein–protein BLAST); when allowing for gaps in both the query and the subject, the closest match was RNVPANFNEAKY-IAF (human and mouse metabotrophic glutamate receptor). To increase its antigenicity, we prepared several BVFs (see the Materials and methods section).

We then tested whether the BVFs could affect obesity. We surmised that the conformational human ApoB100 epitope mimicked by pB1 is conserved in mice (which was later directly confirmed, see Figure 3b). First, juvenile ICR mice were injected with the appetite booster gold-thioglucose (GTG) [22], shifted to an HFD and injected with vehicle or B4-OVA, a BVF produced by chemically coupling a B1 tetramer with ovalbumin. A control group remained on chow. After 12 weeks, the average weight gain of vehicle-treated HFD/GTG mice exceeded the weight gain of chow-fed mice by 24.3 g (40.6–16.3 g) (Table 1, experiment 1). In contrast, the average weight gain of HFD/GTG/B4-OVA mice exceeded the weight gain of chow-fed mice by only 8.5 g (24.8–16.3 g), i.e. B4-OVA inhibited obesity by 65% [100×(24.3–8.5)/24.3].

**Figure 1:**
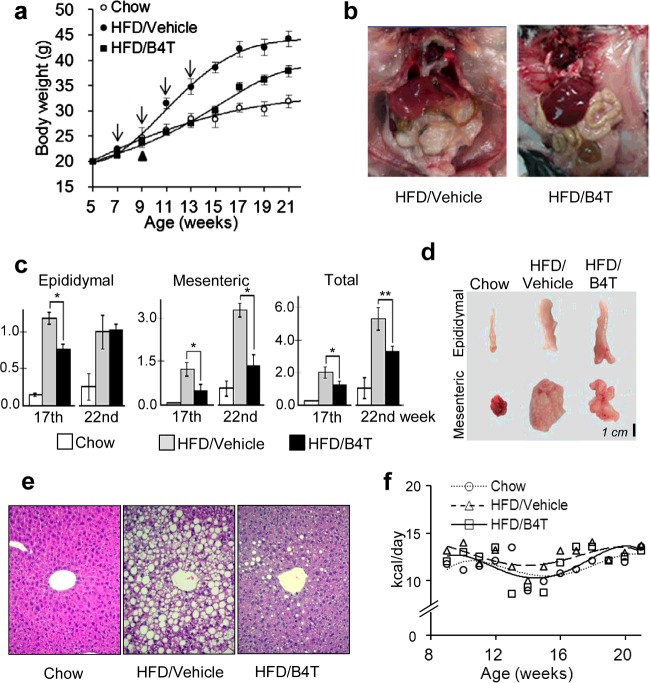
B4T (an ApoB100 mimotope-based vaccine-like formulation) prevents obesity in mice (**a**) Body-weight curves: male C57BL/6 mice were maintained on chow (‘chow’, *n*=6) or switched to a 60% fat diet (HFD) at 9 weeks of age (*n*=18). The diet switch is marked with a black arrowhead. Already at 7 weeks of age, the mice that were destined to receive the HFD were divided into three equal groups: at 7, 9, 11 and 13 weeks of age, one group was injected with vehicle (‘HFD/vehicle’), one group with B4T (‘HFD/B4T’) and one group with B4TB2 (‘HFD/B4TB2’). As the results with B4T and B4TB2 were almost identical, only the B4T version is shown. Injections are marked with arrows. The figure represents the experiments summarized in Table 1. (**b**) Representative photos of viscera at the end of the experiment. Note the reduced visceral fat and the more reddish appearance of the liver in the B4T-treated mouse. (**c**) Fat pad weights determined in week 17 or 22 of the experiment. *P<0.01, **P<0.05 (*n*=5). (**d**) Representative photos of fat pads. (**e**) Prevention of liver steatosis. Representative tissue sections (H&E-stained) are shown. Original magnification ×200. (**f**) Comparisons of daily food intakes.

We then repeated this experiment using modified conditions, now omitting GTG and using C57BL/6 mice as well as other BVFs, B4T and B4TB2, in which the B1 clusters were fused to a helper T-cell epitope (Table 1, experiment 2; Figure 1a). Over 3 months, the average weight gain of the HFD mice exceeded that of the chow mice by 13.3 g (20.7–7.4 g), i.e. the body weight gain attributable to the increased dietary fat was almost twice as high as that attributable to normal growth. At the same time, B4T and B4TB2 inhibited the HFD-induced body-weight increment by 54% and 44%, respectively, and the total body weight increment by 35% and 28% (Table 1). In agreement with the body-weight measurements, the BVF-treated compared with vehicle-treated mice showed a massive reduction in visceral fat that was easily seen with the naked eye (Figure 1b). However, closer inspection showed that different visceral fat depots responded differently: although the dominating mesenteric fat was reduced at both the intermediate and the end-points (weeks 17 and 22), the difference appeared to be transient in epididymal fat (Figures 1c and 1d). Most of the remaining experiments were performed using the BVF version B4T.

We measured some metabolic blood parameters and observed a moderate reduction of total cholesterol, LDL and high-density lipoprotein (HDL) (all *P*<0.05) levels in HFD-fed B4T- compared with vehicle-treated mice, but not down to the level seen in chow-fed mice (see Supplementary Figure S1). The triacylglycerol levels were too variable to see a trend, but were not increased. At the end-point, we also measured non-esterified (‘free’) fatty acids (NEFAs), glucose and glycated haemoglobin (HbA1c) levels (four to eight mice/group). Although NEFA levels were significantly increased in HFD/vehicle compared with chow-fed mice (1.09±0.02 compared with 0.97±0.02 mmol/l; *P*<0.01), NEFA levels in HFD/B4T mice (1.04±0.05 mmol/l) were not significantly different from those in the other groups. Of note, B4T did not normalize glucose levels (97.0±6.9, 194.5±23.3 and 184.7±24.7 mg/dl in chow-fed mice, HFD/vehicle mice and HFD/B4T mice, respectively; *P*<0.001 for chow compared with other diets; no significant difference for HFD/vehicle compared with HFD/B4T). Also, no differences in HbA1c levels were seen (3.49±0.04, 3.46±0.06 and 3.47±0.09% for chow, HFD/vehicle and HFD/B4T mice, respectively).

Histopathological inspection of chow-fed (*n*=3), HFD/vehicle (*n*=4) and HFD/B4T (*n*=6) C57BL/6 mice did not reveal abnormalities in hearts, aortae and kidneys. However, the H&E-stained liver sections of all HFD/vehicle mice studied (*n*=4) exhibited massive centrilobular vacuolation, unambiguously indicating steatosis [23,24]. By contrast, all HFD/B4T livers were clearly less vacuolated, although the degree of vacuolation varied from slight to mild to moderate (two mice each) (Figure 1e). In agreement with the microscopic observations, the livers of the HFD/B4T-treated mice maintained the normal red-brown colour, whereas the livers of the HFD/vehicle-treated mice turned whitish (Figure 1b). Furthermore, B4T treatment blunted an HFD-induced increase of blood transaminase [alanine transaminase (ALT), aspartate transaminase (AST)] levels (*P*<0.01) (see Supplementary Figures S2a and S2b), also indicating an improvement in the liver parenchyma. Creatinine was not altered by either HFD or B4T (see Supplementary Figure S2c). In line with the absence of a non-specific toxicity, neither daily food intakes (Figure 1f) nor growth of the mice (trunk length) (see Supplementary Table S3) was significantly affected. Also, relevant concentrations of B4T lacked non-specific cytotoxicity *in vitro*: although only 0.05 mg of B4T was applied per mouse, up to 0.2–0.4 mg/ml did not reduce the survival of two arbitrarily chosen cell lines, L1210 and human embryonic kidney (HEK)-293T (see Supplementary Figure S2d).

### B4T treatment alters adipocyte metabolism *in vivo*

The large, apparently specific, effect of BVF on fat mass prompted us to look for changes in fat cell metabolism.

**Figure 2:**
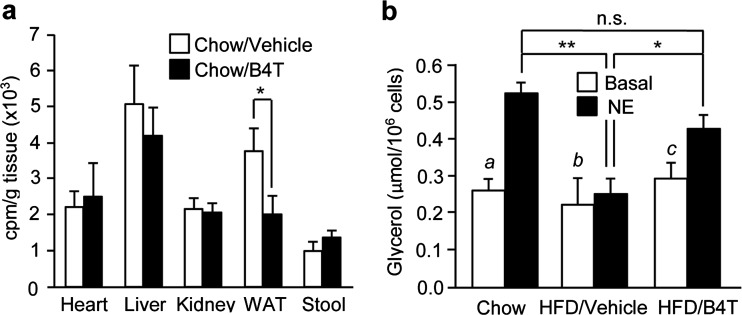
Effects of B4T on adipocyte lipid metabolism *in vivo* and *ex vivo* (**a**) Tissue radioactivities 10 h after an intragastric bolus of glycerol tri[1–^14^C]oleate given to chow-fed rats pre-treated with vehicle or B4T. Two independent experiments: *P<0.01 (*n*=3 rats/group). WAT, white adipose tissue. (**b**) Glycerol release by primary adipocytes *in vitro*, measured in the absence (basal) or presence (NE) of noradrenaline. Adipocytes were isolated from epididymal fat pads of chow-fed, HFD/vehicle or HFD/B4T mice at 21 weeks of age. Three independent experiments: *P<0.05; **P<0.02; *a*, *P*<0.01; *b*, n.s.; *c*, *P*<0.02 (basal compared with NE) (*n*=5–6 mice per group).

We first measured the deposition of an intragastric bolus of glycerol tri[1-^14^C]oleate in tissues of B4T- compared with vehicle-injected rats. Preliminary tests revealed that isotope incorporations into multiple organs peaked ∼10 h after bolus ingestion. At that time, lipid deposition was lower in white adipose tissue and possibly liver (where the decrease was not significant) of B4T- compared with vehicle-injected rats, whereas no change was observed in the heart, kidney and stool (Figure 2a).

Next, we assessed lipolysis in primary adipocytes from HFD/B4T, HFD/vehicle and chow-fed mice. Basal glycerol release was not significantly different between the adipocyte populations. However, unlike adipocytes from chow-fed mice, adipocytes from HFD/vehicle-treated mice had largely lost the ability to respond to noradrenaline stimulation, whereas the responsiveness was largely maintained in the B4T-treated mice (Figure 2b).

### B4T triggers a humoral immune response towards ApoB100

As a result of the discovery of pB1 as an ApoB100 mimotope, we suspected that a BVF-induced anti-ApoB100 immune reaction led to the anti-obesity effect. Such a mechanism would also be in line with the injection schedules and the fact that pB1 and the helper cell epitopes (T-cell or ovalbumin) were individually ineffective (results not shown).

**Figure 3:**
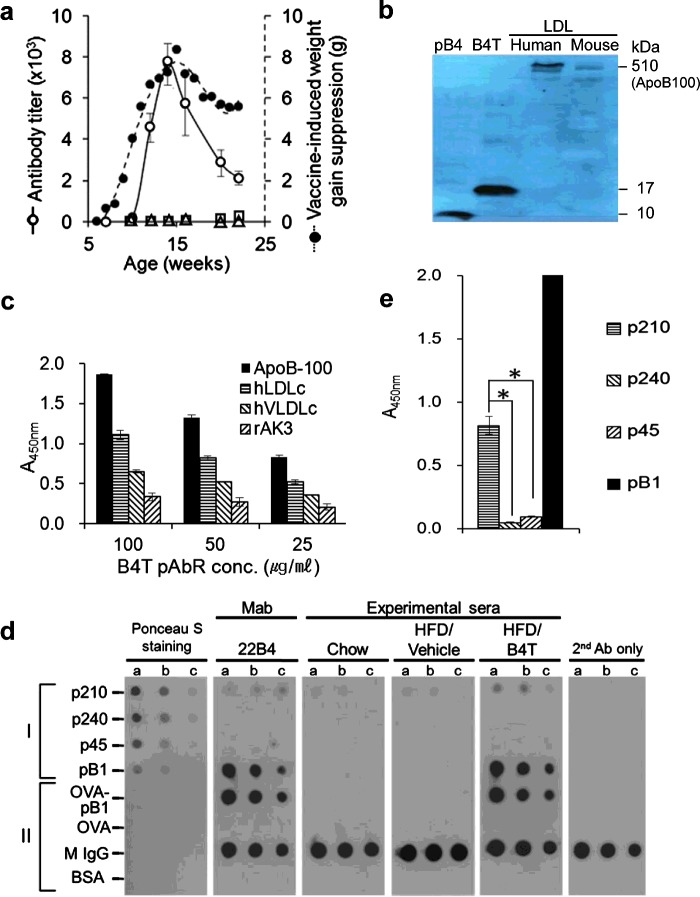
Immunogenicity and immune reactivity of BVFs (**a**) Correlation between B4T-induced antibody titres (determined by ELISA; open circles) and B4T-induced weight gain suppression (filled circles) calculated from the experiment of Figure 1(a) as the differences between weights of HFD/B4T and HFD/vehicle mice at the indicated time points. Also shown are the titres of non-immunized HFD/vehicle controls (open squares) and chow-fed mice (open triangles). Similar results were obtained with HFD/B4TB2 mice. Note that the ELISAs were performed using B4 rather than B4T. (**b**) Western blot revealing molecular target and cross-species reactivity of 22B4, a monoclonal antibody that was induced with B1-OVA: 100 ng of peptide pB4 or B4T or of LDL preparations from the indicated species were loaded on to the gel; the blots were incubated with 1 μg/ml 22B4. Similar results were obtained with polyclonal antibodies raised against B4T in mouse (B4T-pAbM) and rabbit (B4T-pAbR). (**c**) ELISA quantifying reactivity of B4T-pAbR (25, 50 and 100 μg/ml) with 1 μg of human ApoB100, human LDL, human VLDL or the unrelated control protein rAK3. Note that the lower absorbances for VLDL may be due to their larger particle size. (**d**) Dot blots showing reactivities of 22B4 (diluted 1:500) or different sera (diluted 1:200) from mice (chow-fed mice, HFD-fed vehicle-treated mice or B4T-treated HFD-fed mice) with synthetic peptides (p210, p240 and p45) that correspond to different regions of ApoB [25] and with peptide B (here: B1, for monomeric). The sera were harvested at the end of the feeding experiments (21 weeks of age). a, b and c indicate the amounts of the peptides (I) or proteins (II) spotted per well (I: a, b and c=5, 2.5 and 1 μg; II: a, b and c=10, 5 and 2.5 ng for mouse IgG; and a, b and c=100, 50 and 2.5 ng for the other proteins respectively). Similar results were obtained with pooled mouse sera and sera from individual mice. The blot represents five experiments. (**e**) ELISA of the same four peptides and pB1 (100 ng per well) with antibody 22B4 (dilution 1:1000). Two experiments, each in duplicate. *P<0.001.

We therefore first determined whether BVF caused an immune response. Anti-B4T antibodies were indeed induced, and their titres approximately paralleled the B4T-caused weight decrements, whereas very little immune reactivity was detected in HFD/vehicle and chow-fed mice (Figure 3a). We then tested whether the antibodies would also react with ApoB100. Western blotting confirmed that 22B4 (a monoclonal antibody raised against B4T) (Figure 3b), as well as B4T-induced purified mouse and rabbit antibodies (results not shown), recognized not only B4 and B4T, but also human and mouse ApoB100. Hence, the same antibody (22B4) was able to recognize both the mimotope peptide and ApoB100, and this dual reactivity was not exceptional. Moreover, ELISA demonstrated reactivity against native human ApoB and partially purified LDL and VLDL, whereas the unrelated recombinant adenylate kinase isoenzyme 3 (rAK3) was non-reactive (Figure 3c). The apparent reactivity with VLDL was lower than with LDL, probably due to the larger particle size of the VLDL.

To identify an epitope, we performed dot-blot experiments with synthetic peptides from a published set of peptides [25], including p45 from the N-terminal half of ApoB100 (amino acids 661–680), and p210 (3136–3155) and p240 (3586–3605) from the C-terminal half. The antibody 22B4 reacted not only with the immunizing mimotope (spotted in its monomeric form, pB1), but also with p210, whereas low or no reactivity was observed with p240 and p45 (Figure 3d). A low background reactivity was exhibited by serum from chow-fed and HFD-fed, vehicle-treated mice, whereas a clear reaction with p210 (but not p240 or p45) was seen with HFD-fed B4T-treated mice. The dot-blot results were further supported by ELISA, demonstrating that 22B4 reacted far better with p210 than p240 or p45 (Figure 3e). Using ELISA against human ApoB100, we determined the apparent dissociation constant, *K*_d_, of B4T-pAbR (3.11–7.12×10^−7^/M) as approximately 1/100th of the *K*_d_ for 6H12 (1.22×10^−9^/M).

We also found that pB4T predominantly induced IgG2b and IgG1 antibodies (see Supplementary Figure S3a) and a T-helper (Th) 2-type cytokine spectrum (see Supplementary Figures S3b and S3c), which is characteristic of a humorally biased immune response [26].

Thus, B4T induced a humoral immune response towards native ApoB100 of both LDL and VLDL, and targeted a specific epitope in its C-terminal half.

### The ApoB100-directed immune response modulates functions of LDL

ApoB100 is known to mediate two LDL functions, endocytic lipid delivery and adipocyte lipolysis inhibition, both through interaction with an LDL receptor (LDLR) [1,27]. We used these functions to assess whether and how the mimotope-induced antibodies can modulate the lipid-metabolic functions of ApoB100.

**Figure 4:**
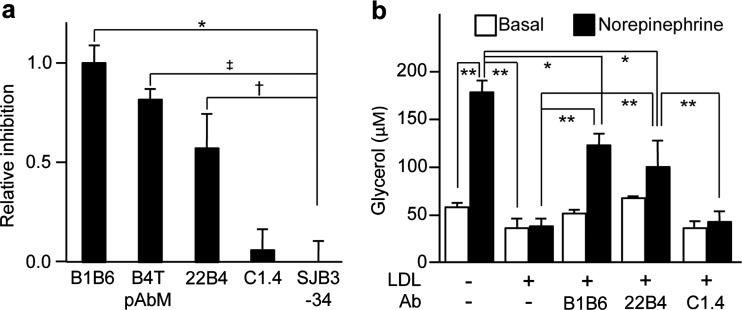
Mimotope-induced antibodies modulate functions of LDL *in vitro* (**a**) Effect on DiI-LDL uptake by cultured 3T3L1 adipocytes. Results are normalized to the inhibition caused by antibody (Ab) B1B6 (absolute inhibitions caused by B1B6 ranged from 36% to 64%). The figure represents three experiments; *P<0.001, ‡*P*<0.005, †*P*<0.01 (*n*=9). (**b**) Antibody effects on basal and noradrenaline-stimulated lipolysis (release of glycerol) by 3T3L1 adipocytes. The figure represents three experiments. *P<0.05, **P<0.005 (*n*=5). (See Supplementary Table S1 for description of the antibodies.)

As shown in Figure 4(a), the polyclonal antibody B4T pAbM and the monoclonal antibody 22B4 blocked the uptake of DiI-labelled human LDL by 3T3L1 adipocytes [16]. The effect was comparable in magnitude to that of a commercial antibody (B1B6) directed against the LDLR recognition site in the C-terminal half of ApoB100, whereas an antibody (C1.4) directed against the N-terminal half was as ineffective as the unrelated SJB3-34 (Figure 4a). As the B4T-induced monoclonal antibody 22B4, as well as the sera of HFD/B4T-treated mice, reacts specifically with the C-terminal half of ApoB100 (Figures 3d and 3e), the peptide B-induced antibodies most probably interfere with the lipoprotein uptake by adipocytes through binding to the C-terminal half of ApoB100.

Next, we studied the potential influence of mimotope-induced antibodies on 3T3L1 adipocyte lipolysis (Figure 4b). As previously shown [27], LDL significantly inhibited noradrenaline-stimulated glycerol release in 3T3L1 adipocytes. Importantly, the effect was partially but significantly prevented by antibodies B1B6 (raised against a peptide in the C-terminal half of ApoB100) and 22B4 (raised against pB1), but not by antibody C1.4 (raised against a peptide in the N-terminal half of ApoB100). These effects on lipolysis, as assessed by glycerol release, were paralleled by changes in the forward scatter values in FACS runs (see Supplementary Figure S4), indicating lipolysis-related changes in cell size.

### The ApoB100-directed immune response facilitates the uptake and metabolism of native LDL by macrophages

**Figure 5:**
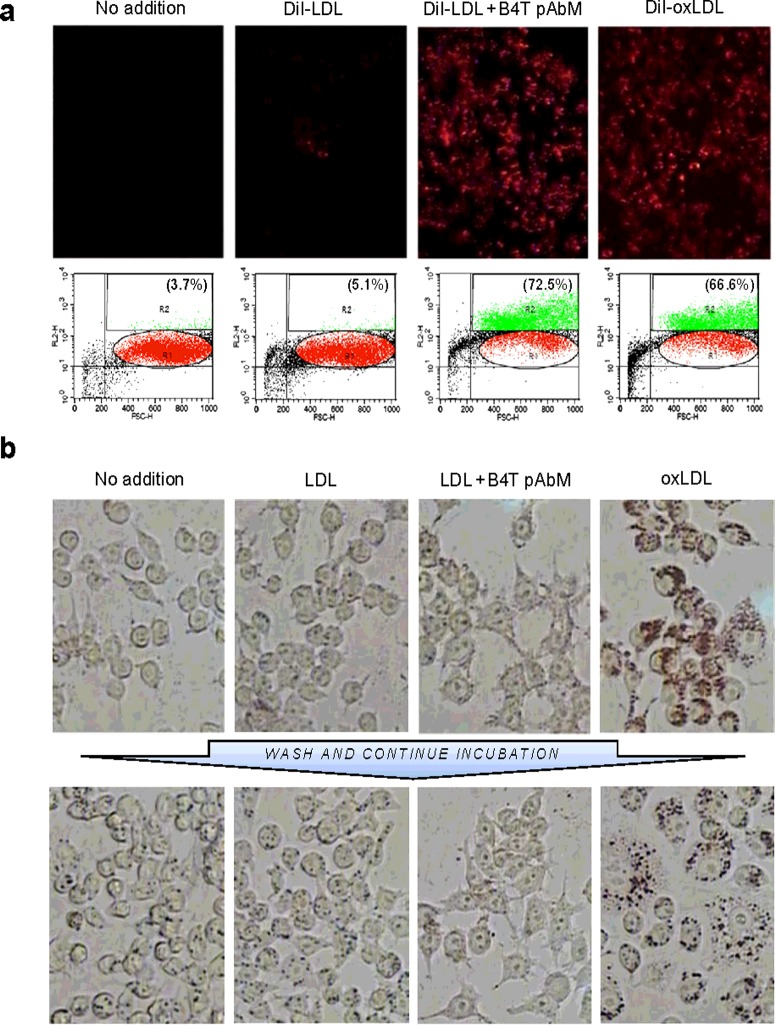
Effect of B4T pAbR on native LDL uptake by RAW264.7 macrophages *in vitro* (**a**) Uptake of DiI-labelled lipoproteins. Cells were incubated for 24 h without addition, with native DiI-LDL, DiI-LDL pre-incubated with antibody p4T-pAbM, or DiI-oxLDL. Upper row shows fluorescence photos of the respective cell cultures; lower row shows flow cytometry after trypsinization: *x*-axis is FSC-H (forward scatter), *y*-axis is FL2-H (fluorescence); gate R1 (red) represents the normal macrophage population, and gate R2 (green) represents the population of the macrophages that engulfed lipid particles. The figure represents three experiments. (**b**) Anti-B4T-mediated LDL uptake does not cause foam cell formation. The macrophages were first incubated as in (**a**), but using non-labelled lipoproteins. They were either stained with Oil Red O (upper row) or washed and incubated in fresh medium without additions for another 24 h, and then stained with Oil Red O (lower row). The figure represents three experiments. Original magnification ×200.

As shown above, the BVF-induced antibody spectrum was biased towards IgG2b and IgG1, which are good opsonins [28,29]. We therefore assessed the effect of B4T-pAbR on the phagocytosis of LDL particles with the murine macrophage cell line RAW264.7. After addition of DiI-labelled native LDL (DiI-LDL), weak fluorescence was seen in only a few cells, but when the DiI-LDL were pre-incubated with B4T-pAbM, many cells exhibited strong fluorescence (Figure 5a, upper row). By contrast, addition of DiI-labelled oxLDL (DiI-oxLDL) led to a strong fluorescence without the antibody (Figure 5a, upper row). In fact, the proportions of labelled cells (as assessed by FACS analysis) were similar (approximately two-thirds of the total number) when using antibody-incubated DiI-LDL or free DiI-oxLDL (Figure 5a, lower row). In equivalent experiments using unlabelled lipoproteins, we stained the cells with Oil Red O. Analogous to the results obtained with the DiI label, there was little if any Oil Red O staining after incubation with (unlabelled) native LDL, but strong staining of most of the cells after incubation with oxLDL (Figure 5b, upper row). The cells also underwent a morphological change, characterized by enlargement and granulation. In contrast with the DiI fluorescence, however, incubation with antibody-incubated (unlabelled) native LDL led to an albeit visible but just modest Oil Red O staining and a less pronounced morphological change (Figure 5b, upper row). After washing out the additives and incubating for another 24 h, the oxLDL-treated cells retained much of the Oil Red O-stainable material and assumed the characteristic foam cell morphology, whereas the other treated variants were essentially indistinguishable from the untreated control (Figure 5c, lower row).

## DISCUSSION

### A mimotope of a C-terminal epitope of ApoB100 can prevent obesity

Although ApoB100 has long been recognized as an important player in peripheral fat deposition, there is no firm evidence that it exerts specific control over body weight in normal conditions or obesity. Therefore, ApoB100 is not considered a promising molecular target in anti-obesity approaches. However, we showed in the present study that, in mice, vaccine-like formulations that are based on a peptide (pB1) mimicking an epitope in the C-terminal half of ApoB100 (i.e. not in ApoB48) prevented HFD-induced obesity. Our findings thus suggest an ApoB100-based immunization approach against obesity.

### Mechanisms underlying the anti-obesity effect

As we discovered pB1 by using an anti-ApoB100 antibody, it was natural to ask whether the BVFs (B4T in most experiments) exerted their anti-obesity effect through antibodies that modulate the functions of ApoB100. Indeed, when we injected the BVFs in a typical immunization schedule, they induced antibodies that specifically recognized ApoB100 in Western blots and reacted with native ApoB100 in ELISAs; importantly, the antibody titres correlated with the weight decrements in HFD-fed/immunized mice. As discussed in the following paragraphs, our *in vitro* data suggest that, at least in part, the antibodies cause their effects on lipid metabolism by blocking the interaction between ApoB100 and LDLRs, and by acting as opsonins for the ApoB100-containing lipoproteins.

The antibodies induced by B4T or B4-OVA, as well as a C-terminally acting commercial anti-ApoB antibody, but not an N-terminally acting antibody, blunted the uptake of LDL-associated lipids by cultured 3T3L1 adipocytes. Furthermore, in addition to the natural ApoB100, a synthetic peptide (p210) from the C-terminal half of ApoB100 was recognized by a pB1-induced monoclonal antibody (22B4) as well as by serum from HFD/B4T-treated mice, whereas peptides from other regions of ApoB100 were not recognized. These results indicate that the pB1-induced antibodies target the C-terminal half of ApoB100, which is known to bind the LDLR [30]. The possibility that these antibodies interfere with LDLR binding is also consistent with our observation that pB1-induced antibodies blunted the inhibitory effect of LDL on adipocyte lipolysis *in vitro*, which is known to be mediated through the LDLR [27]. It is of interest that the effect of this relatively brief antibody treatment of the 3T3L1 cells was comparable in magnitude to the long-term effect of immunization on glycerol release by freshly isolated adipocytes (Figures 2b and 4b). Hence, direct antibody inhibition of LDLR-mediated inhibition of the lipolysis may contribute to the anti-obesity effect *in vivo*, which might be further enhanced [27] by the (for whatever reason) slightly reduced LDL serum concentrations in HFD/B4T-treated mice. Compared with lipolysis, the observed reduced LDL uptake is less likely to be important *in vivo*, because lipoprotein uptake is not thought to be a major determinant of adipocyte fat mass [3]; nevertheless, the LDL uptake experiment provides independent support for the same molecular mechanism (antibody-mediated inhibition of the ApoB100–LDLR interaction) that we propose as underlying the activation of lipolysis.

It seems questionable, however, that the increased lipolysis due to inhibited ApoB100–LDLR binding can fully explain the prevention of obesity. To our knowledge, mutations of ApoB that block interaction with LDLR [31] have not been described as preventing obesity. Also, the homoeostatic capacity of the fat [10] suggests that additional mechanisms are involved. In this respect, we noticed an antibody-mediated increase of LDL uptake by cultured macrophages. The increase was dramatic when assessed with the fluorescent label DiI, but moderate when judged by Oil Red O staining. This discrepancy suggests that, unlike the artificial and stable membrane dye DiI [32], the native LDL-associated natural lipids were metabolized rapidly, at least compared with oxLDL, thus preventing the foam cell formation seen with oxLDL. We also found that the B4T-induced antibodies were predominantly of the IgG isotype, and it is well known that macrophages take up IgG-bound LDL via Fc receptors and catabolize the associated lipid [33]. Whether foam cell formation then occurs will generally depend on the balance of lipid deposition and degradation; in our model breakdown appears to prevail and might play an important role in the anti-obesity effect of B4T. In fact, it is known that adipose tissue macrophages are capable of active fatty acid oxidation [34] and accumulate substantially during obesity, with contributions as high as 50% of cell numbers in the adipose tissue [35]. If this mechanism holds true, it would raise questions about the participating macrophages, which can assume more (anti-inflammatory) and less (pro-inflammatory) oxidative states [36]. Note also that the mimotope-induced antibodies reacted with both LDL and VLDL (Figure 3c), hence the opsonization mechanism may not be limited to LDL (in contrast to the effect on lipolysis; compare Skogsberg et al. [27]).

By removing lipid from the circulation, the macrophage mechanism might help in understanding how B4T prevents fatty liver formation. It is also possible that antibody binding prevented the activation of hepatic lipase, which interacts with the C-terminal portion of ApoB100 [37]. In any case, it is unlikely that the B4T effect on the liver is secondary to that on adipose tissue or vice versa, because selective inhibition of lipid deposition in one of the tissues should favour the opposite effect on the other. Note, in this context, that genetic deficiencies of human and murine ApoB usually lead to a fatty liver [9,10], which is the opposite of what we observe. Fatty liver is caused by defective hepatic lipoprotein assembly/secretion, suggesting that it is an advantage of our approach that it acts on normal, already secreted lipoproteins.

Finally, we cannot rule out that increased muscle activity contributed to the weight reduction, although such a mechanism seems implausible. We did not observe a conspicuous change in physical activity, but quantitative measurements are needed to settle this point.

### Human relevance

The present study revealed a robust anti-obesity effect of B4T and other BVFs in mice; however, we anticipate that B4T will be even more effective in humans. First, peptide pB1 is likely to match a human epitope better because it was discovered by using anti-human ApoB100 antibodies; indeed, our anti-pB1 antibodies reacted stronger with human than with mouse LDL (Figure 3b). Secondly, antibodies raised by BVFs react with an epitope present in ApoB100 but not ApoB48, and ApoB100 is much more dominant than ApoB48 in human livers compared with rodent livers [2]. In contrast, the BVFs should not be of direct relevance to gut-derived lipoproteins, which are dominated by ApoB48 in both humans and mice [2]. It should be interesting to see how our immunization approach performs in rabbit, which exhibits a more human-like lipoprotein profile than mouse [4].

So far, we did not see adverse effects of B4T. It did not exhibit direct cytotoxicity on cultured L1210 and HEK-293T cells at life-like concentrations. It inhibited neither appetite nor growth and did not cause histological changes except in the liver, where the effect was actually beneficial (prevention of fatty liver). It is also unlikely that B4T is pro-atherogenic because: (i) p210 (which is recognized by anti-pB1 antibodies) has recently been used for immunization against atherosclerosis [38], and (ii) in our *in vitro* experiments with macrophages, the antibody-induced increase of lipid uptake did not cause foam cell formation (unlike oxLDL). Of course, a deeper risk analysis would be needed, not only with respect to atherosclerosis, but also as altered ApoB levels have been associated with neuropathology [39,40] and infection susceptibility [41]. Finally, the fact that we did not observe a normalized glucose level may limit potential applications of B4T. Conceivably, the increased lipolysis counteracts improvements of glucose metabolism [42], suggesting that B4T might perform best in combination with other drugs.

### Conclusion

Our data suggest the following scenario: an artificial peptide that mimics an epitope in the C-terminal half of human ApoB100 induces IgG antibodies, which exert at least two effects. (i) The antibodies bind specifically to ApoB100 and interfere with its effects on adipocyte lipid metabolism, leading to enhanced lipolysis and possibly reduced lipid uptake. Given the homoeostatic capacity of fat tissue [10], it remains to be seen whether this effect alone can affect fat mass, and future work will need to more comprehensively assess the effect of immunization on adipocyte fat metabolism. (ii) Via opsonization, the ApoB100-bound antibodies also bind to macrophages, causing them to take up ApoB-associated lipid (without creating foam cells). Thus, the antibodies may divert excessive fat to the most abundant non-adipocyte cell type known to accumulate in the fat tissue during obesity [35]. Clearly, the fat-oxidative capacity of macrophages deserves further study. Regardless of which mechanism(s) turn(s) out to be most significant, our data for the first time raise the possibility that ApoB100 may become a direct molecular target in the therapy of HFD-induced obesity.

## Abbreviations

ALT: alanine transaminase
ApoB: apolipoprotein B
AST: aspartate transaminase
BVF: pB1-based vaccine-like formulation
DiI: 1,1′-dioctadecyl-3,3,3′,3′-tetramethyl-indocarbocyanine percholate
DMEM: Dulbecco's modified Eagle's medium
GTG: gold-thioglucose
H&E: haematoxylin and eosin
HbA1c: glycated haemoglobin
HDL: high-density lipoprotein
HEK: human embryonic kidney
HFD: high-fat diet
HRP: horseradish peroxidase
IDL: intermediate-density lipoprotein
Ig: immunoglobulin
LDL: low-density lipoprotein
LDLR: LDL receptor
NEFA: non-esterified (‘free’) fatty acid
OVA: ovalbumin
oxLDL: oxidised LDL
rAK3: recombinant adenylate kinase isoenzyme 3
VLDL: very-low-density lipoprotein
